# RSC Chromatin-Remodeling Complex Is Important for Mitochondrial Function in *Saccharomyces cerevisiae*

**DOI:** 10.1371/journal.pone.0130397

**Published:** 2015-06-18

**Authors:** Yuko Imamura, Feifei Yu, Misaki Nakamura, Yuhki Chihara, Kyo Okane, Masahiro Sato, Muneyoshi Kanai, Ryoko Hamada, Masaru Ueno, Masashi Yukawa, Eiko Tsuchiya

**Affiliations:** 1 Department of Molecular Biotechnology, Graduate School of Advanced Sciences of Matter, Hiroshima University, 1-3-1 Kagamiyama, Higashi-Hiroshima, Hiroshima, 739–8530, Japan; 2 National Research Institute of Brewing, 3-7-1 Kagamiyama, Higashi-Hiroshima, Hiroshima, 739–0046, Japan; University of Tokyo, JAPAN

## Abstract

RSC (Remodel the Structure of Chromatin) is an ATP-dependent chromatin remodeling complex essential for the growth of *Saccharomyces cerevisiae*. RSC exists as two distinct isoforms that share core subunits including the ATPase subunit Nps1/Sth1 but contain either Rsc1or Rsc2. Using the synthetic genetic array (SGA) of the non-essential null mutation method, we screened for mutations exhibiting synthetic growth defects in combination with the temperature-sensitive mutant, *nps1-105*, and found connections between mitochondrial function and RSC. *rsc* mutants, including *rsc1Δ*, *rsc2Δ*, and *nps1-13*, another temperature-sensitive *nps1* mutant, exhibited defective respiratory growth; in addition, *rsc2Δ* and *nps1-13* contained aggregated mitochondria. The *rsc2Δ* phenotypes were relieved by *RSC1* overexpression, indicating that the isoforms play a redundant role in respiratory growth. Genome-wide expression analysis in *nps1-13* under respiratory conditions suggested that RSC regulates the transcription of some target genes of the HAP complex, a transcriptional activator of respiratory gene expression. Nps1 physically interacted with Hap4, the transcriptional activator moiety of the HAP complex, and overexpression of *HAP4* alleviated respiratory defects in *nps1-13*, suggesting that RSC plays pivotal roles in mitochondrial gene expression and shares a set of target genes with the HAP complex.

## Introduction

In eukaryotes, chromatin structure remodeling plays crucial roles in various nuclear processes, including transcription, DNA replication, repair, and recombination. Two general classes of enzymes that regulate chromatin remodeling are as follows: enzymes that covalently modify histone molecules and enzymes that alter nucleosome structures using energy from ATP hydrolysis. These enzymes are highly conserved in eukaryotes. ATP-dependent chromatin remodeling factors can be further divided into four groups, SWI/SNF, Ino80/SWR, ISWI, and CHD complexes, based on characteristics of the ATPase subunits molecular structures in each complex. Out of these, SWI/SNF complexes are known to be tumor suppressors in mammalian cells (reviewed in [1] and [2]). Therefore, insights into the functions of this SWI/SNF-type complex will facilitate a better understanding of the role of chromatin remodeling in both DNA-metabolism regulation and cancer formation. In mammalian cells, however, several hundred variant SWI/SNF complexes are thought to possibly exist because of the large number of subunits encoded by their gene families, of which variants differ among cells of different lineages; such variations cause difficulty in the analysis of complex function [1].

*Saccharomyces cerevisiae* possesses the following two SWI/SNF-type complexes: the nongrowth-essential SWI/SNF complex [3, 4] and RSC complex, which is essential for both mitotic and meiotic growth [5–7]. The RSC complex is composed of 17 subunits, and at least two distinct types of complex containing either Rsc1 or Rsc2 are present. Previous studies have shown that RSC functions in a pleiotropic manner to regulate transcription, DNA repair, and chromosome segregation (reviewed in [8] and [9]); however, the scope of RSC function is still enigmatic.

To obtain a more global insight into the role of RSC in cell growth, we performed a synthetic genetic array (SGA) analysis, which comprised a genome-wide screening of synthetic lethality/sickness, using *nps1-105*, a temperature-sensitive mutant allele of the *NPS1/STH1* gene that encodes the ATPase subunit of RSC, as a query. Using this screening procedure, we determined that RSC played pivotal roles in mitochondrial function. A part of this RSC function was achieved via the action of HAP complex, a transcription factor composed of Hap2, Hap3, Hap4, and Hap5 that plays an essential role in respiratory gene expression [10].

## Materials and Methods

### Strains and culture conditions

All strains were isogenic to BY4741 (MATa *his3Δ1 leu2Δ0 met15Δ0 ura3Δ0*). The yeast strains used in this study are listed in Table 1. Standard procedures were used for mating, sporulation, transformation, and tetrad dissection. All media were prepared as described previously [11]. Because our *rsc2Δ* haploid strain frequently bore diploids, possibly due to some chromatin defect, we constructed homozygous diploids for the *rsc* mutation-bearing strains used in this study. The homo-diploid of null mutations for *RSC1*, *RSC2*, and *RSC7* was constructed by the transformation of the HO endonuclease gene on a plasmid (YEp13*HO*), using standard methods [12]. Alleles of *nps1-105* and *nps1-13* were described previously [13, 14]. To construct *HAP4-HA*, *NPS1-TAP HAP4-HA*, and *nps1-13 HAP4-HA*, we designed primers to introduce the 6 HA sequence in frame with the C-terminus of the *HAP4* gene, followed by a *CYC* terminator and *URA3* gene. PCR reactions were performed with each primer pair, using the plasmid pBS*6HA-URA3* as the template; appropriate strains were transformed with the resulting DNA fragments. Correct insertion was verified by sequencing. All primer sequences for PCR reactions are listed in Table 2. Cells were grown at 28°C in YPD medium (1% yeast extract, 2% peptone, 2% glucose), YPEG medium (1% yeast extract, 2% peptone, 3% ethanol, 3% glycerol) or YPL medium (1% yeast extract, 2% peptone, 2% lactic acid, pH 5.5 adjusted with NaOH). Spot assays were performed by spotting 5–10 μl of cells at a concentration of 1 10^7^ cells/ml after 5-fold serial dilutions onto YPD or YPEG plates. The plates were incubated at various temperatures from 30°C to 35°C as necessary.

**Table 1:** Strains used in this study.

Strain		Genotype	Source
BY4743		*MATa/α his3Δ1/his3Δ1 leu2Δ0/leu2Δ0 ura3Δ0/ura3Δ0 MET15/met15Δ0 LYS2/lys2Δ0*	Research Genetics
BY4741		*MATa his3Δ1 leu2Δ0 ura3Δ0 met15Δ0*	Research Genetics
BY-1G	*nps1-105*	*MATα nps1-105-TAP*::*LEU2 can1Δ*::*MFA1pr-HIS3-MFα1pr-URA3 his3Δ1 leu2Δ0 ura3Δ0 lys2Δ0*	This study
BY-1F	*nps1-13*	*MATa nps1-13 his3Δ1 leu2Δ0 ura3Δ0 met15Δ0*	This study
BYI-1	*rsc1Δ*	*MATa/α rsc1Δ*::*KanMX4/rsc1Δ*::*KanMX4 his3Δ1/his3Δ1 leu2Δ0/leu2Δ0 ura3Δ0/ura3Δ0 met15Δ0/met15Δ0*	This study
BYI-2	*rsc2Δ*	*MATa/α rsc2Δ*::*KanMX4/rsc2Δ*::*KanMX4 his3Δ1/his3Δ1 leu2Δ0/leu2Δ0 ura3Δ0/ura3Δ0 met15Δ0/met15Δ0*	This study
BYI-3	*nps1-13*	*MATa/α nps1-13/nps1-13 his3Δ1/his3Δ1 leu2Δ0/leu2Δ0 ura3Δ0/ura3Δ0 met15Δ0/met15Δ0*	This study
BYI-7	*NPS1-TAP*	*MATa NPS1-TAP-KanMX4 his3Δ1 leu2Δ0 ura3Δ0 met15Δ0*	[15]
BYI-17	*nps1-105*	*MATa/α nps1-105/nps1-105 his3Δ1/his3Δ1 leu2Δ0/leu2Δ0 ura3Δ0/ura3Δ0 met15Δ0/met15Δ0*	This study
BYI-18	*rsc7Δ*	*MATa/α rsc7Δ*::*KanMX4/rsc7Δ*::*KanMX4 his3Δ1/his3Δ1 leu2Δ0/leu2Δ0 ura3Δ0/ura3Δ0 met15Δ0/met15Δ0*	This study
BYI-19	*HAP4-HA*	*MATa/α HAP4-6HA*::*URA3/ HAP4-6HA*::*URA3 his3Δ1/his3Δ1 leu2Δ0/leu2Δ0 ura3Δ0/ura3Δ0 met15Δ0/met15Δ0*	This study
BYI-20	*NPS1-TAP HAP4-HA*	*MATa NPS1-TAP-KanMX4 HAP4-6HA*::*URA3 his3Δ1 leu2Δ0 ura3Δ0 met15Δ0*	This study
BYI-21	*nps1-13 HAP4-HA*	*MATa/α nps1-13/nps1-13 HAP4-6HA*::*URA3/ HAP4-6HA*::*URA3 his3Δ1/his3Δ1 leu2Δ0/leu2Δ0 ura3Δ0/ura3Δ0 met15Δ0/met15Δ0*	This study
BYI-22	*hap4Δ*	*MATa/α hap4Δ/ hap4Δ his3Δ1/his3Δ1 leu2Δ0/leu2Δ0 ura3Δ0/ura3Δ0 met15Δ0/met15Δ0*	This study

**Table 2:** Primers.

Primers	Sequence
ACT1-RtF	CCAGAAGCTTTGTTCCATCC
ACT1-RtR	CGGACATAACGATGTTACCG
ATP1-RtF	GCCGGTGTTAATGGTCATTT
ATP1-RtR	TAGCACTCTTTAGAGATGCC
ATP16-RtF	AAGCTTTTCCATTGGAATCC
ATP16-RtR	TTGAATTGCAGCTTCTGCGG
COR1-RtF	TCTCTGGGTGAGGCTTTCAA
COR1-RtR	TTCAATTTGGCCTGTACCAG
COX6-RtF	ACCTACCGCAATTAGAGTAT
COX6-RtR	AGCTTGGAAATAGCTCTTCC
COX12-RtF	AAGGGCGAAGATTTTGCTCC
COX12-RtR	TCTGAGTTGATATCACCTGC
HAP4-F	AACAAAGGATCCAAAATGACCGCAAAG
HAP4-R	CGGATACTCGAGAATGCTCTTAGG
HAP4-6HA-F	GACGACCTTGACGAAGATGTCGATTTTTTAAAGGTACAAGTATTTGGATCCTCTAGCTACCCATA
HAP4-6HA-R	TTTCGTGATTTTTAGTTGTTTTCGTTTTATTGCAACATGCCTATTCGAGGTCGACGGTATCGATA

### Plasmids

The plasmids used in this study are listed in Table 3. YEp13*RSC1*-3MYC was constructed as previously [16]. The plasmid pRS426*GPDpr*::*HAP4* was constructed as follows: A DNA fragment containing the *HAP4* ORF (1 to +349) harboring *Bam*HI and *Xho*I sites at the 5’ and 3’ ends, respectively, was generated via PCR using the primers HAP4-F and HAP4-R and genomic DNA as the template. The resulting DNA fragment was subcloned into the corresponding sites of pRS426GPD [17]. The plasmid pBS*6HA-URA3* comprised pBluescript II containing the 6 HA sequence, *CYC* terminator, and *URA3* in that order.

**Table 3:** Plasmids.

Plasmid	Description	Source
YEp13	2μ, *LEU2*	Lab stock
YEp13*HO*	YEp13 containing *HO*	Lab stock
YEp13*RSC1*-3MYC	YEp13 containing *RSC1*-3MYC	[16]
pRS426	2μ, *URA3*	Lab stock
pRS426GPD	2μ, *URA3*, GPD promoter, CYC terminator	[17]
pRS426*GPDpr*::*HAP4*	pRS426GPD containing *HAP4*	This study
pBS6HA-URA3	pBluescript II carrying 6xHA, CYC terminator and *URA3*	This study

### Synthetic genetic array (SGA) analysis

SGA analysis was performed basically as described by Tong [18], with some modification. To allow the selection of both *MATa* and *MATα* double-mutant strains, we integrated the *MFA1pr-HIS3-MFα1pr-URA3* sequence into the *CAN1* locus of *nps1-105-TAP*::*LEU2* (BY-1G). This strain was mated with the yeast haploid deletion set (BY4741 background) from Research Genetics (Invitrogen) on rich media; diploids were selected on synthetic complete (SC) medium containing 500 μg/ml G418 but lacking leucine. These diploids were induced to sporulate, and meiotic haploid *MATa* or *MATα* double mutants were selected on SC medium containing canavanine and G418, but lacking leucine, arginine, and histidine, or on SC medium containing canavanine and G418, but lacking leucine, arginine, and uracil, respectively. To exclude sporulation-deficient mutants caused by haploinsufficiency, we evaluated the growth of meiotic haploid cells via simultaneous selection on haploid-selection medium (SC-His-Arg+Canavanine or SC-Ura-Arg+Canavanine). To evaluate synthetic lethality/sickness interactions with *nps1-105*, we selected a *his3Δ nps1-105* haploid double mutant as a control query each time and compared the growth level of each haploid double mutant strain with that of the control strain by visual inspection. Double mutants were categorized into three groups according to their growth levels (normal, slow, and no growth) at 28°C. We performed another SGA analysis to confirm the growth levels of the double mutants, which exhibited slow or no growth on both or either mating-type background in our first SGA screening. To strictly confirm reproducibility, we confirmed the growth levels of eight double-mutant progenies (*MATa* 4, *MATα* 4) selected independently from the same parental heterozygous diploid per allele (S1 Table). We selected alleles for which all double-mutant progenies exhibited slow or no growth as those exhibiting synthetic lethality/sickness interactions with *nps1-105*.

### Microscopic analysis

Cells were grown to log phase, washed with HEPES buffer (10 mM HEPES-KOH, pH7.4, containing 5% glucose), and stained with 50 nM Mito-Tracker (Molecular Probes) for 10 min in the dark to visualize mitochondria. To detect intracellular reactive oxygen species (ROS), cells were incubated with 5 μg/ml dihydroethidium (Sigma-Aldrich) for 20 min in the dark. The stained cells were observed under a fluorescence microscope (Olympus BX51).

### DNA microarray analysis

Microarray analysis was performed as described previously [19, 20], using the Gene Chip Yeast Genome 2.0 Array (Affymetrix). For RNA preparation, wild-type (WT; BY4743) and *nps1-13* (BYI-3) cells pre-grown in YPD medium were inoculated in YPEG medium at a concentration of 1 10^6^ cells/ml and grown to mid-log phase for 4 h. Biotinylated cRNA was prepared from 500 ng of total RNA according to the standard Affymetrix protocol, and 5 μg of cRNA was hybridized for 4 h at 45°C on the GeneChip Yeast Genome 2.0 Array. GeneChips were washed and stained using the Hybridization, Wash, and Stain Kit (Affymetrix). Data were analyzed with Operating Software (GCOS) v1.4, using the Affymetrix default analysis settings and global scaling as the normalization method. The trimmed mean target intensity of each array was arbitrarily set to 500. A given gene was considered induced or repressed when the expression ratio was, respectively, higher or lower than 2.0. Microarray data can be retrieved from Gene Expression Omnibus (GEO) under the accession code GSE66685.

### Gene ontology term enrichment analysis

To identify enriched Gene Ontology (GO) terms, we used the *Saccharomyces* Genome Database Gene Ontology Slim-Mapper (http://www.yeastgenome.org/cgi-bin/GO/goSlimMapper.pl). To evaluate the significance of GO term enrichment among genes that deletions are responsible for growth defects in combination with *nps1-105* or among genes that expression is significantly increased or decreased in *nps1-13*, we performed a hypergeometric distribution. *P*-values represent the probability that the given list of genes intersects with any functional category occurs by chance. To test HAP-regulated gene enrichment, we referred to the gene set that transcription level was higher in WT cells than in the *hap2Δ* and *hap4Δ* mutants [21].

### RNA preparation and quantitative real-time PCR analysis

Total RNA was purified using an RNeasy MiniKit (Qiagen) according to the manufacturer’s instructions. Quantitative real-time RT-PCR was performed using a One Step SYBR PrimeScript RT-PCR Kit II (TaKaRa) and a Light Cycler (Roche Applied Science). Primers for the specified genes were validated with standard curves before use. Transcript abundance was normalized to *ACT1* transcripts. The PCR primers used in this study are listed in Table 2.

### Immunoblotting

Yeast cells were grown to log phase, and cell lysates were thereafter prepared at the appropriate times. Proteins in each cell lysate were resolved by SDS-PAGE, followed by immunoblotting, or processed for immunoprecipitation as described previously [14]. The intensities of protein bands obtained by immunoblotting were measured using the image analyzing software, Image-J (NIH, USA). The following antibodies were used: anti-Cdc28 (Santa Cruz Biotechnology, Inc), anti-HA (Covance), and anti-TAP (Open Biosystems).

## Results and Discussion

### 1. Screening of null mutations indicated a synthetic growth defect in combination with *nps1-105*

To understand the scope of functions of RSC complex functions, we performed an SGA analysis by crossing a temperature-sensitive mutant of ATPase subunit, *nps1-105*, with a collection of 4,847 viable deletion strains. Screening was performed three times, and reproducible candidates were further analyzed by tetrad analysis. As shown in Table 4, 95 gene deletions exhibited either synthetic lethal or slow growth phenotypes in combination with the *nps1-105* mutation. Among these genes, 18 overlapped with those previously identified by an SGA screening using *rsc7Δ* as a query [22]. These 95 genes were categorized into the following five broad classes according to their involvement: (1) chromosome metabolism, (2) translation, (3) mitochondria, (4) general metabolism, and (5) transport. In fact, our *Saccharomyces* Genome Database GO Slim-Mapper-based analysis revealed that these 95 genes were significantly enriched with respect to GO terms related to these five broad functional classes (S2 Table). The chromosome metabolism class included members of the Ino80 chromatin-remodeling complex, transcription initiation and elongation complexes, spindle assembly checkpoint, and RNA processing. The deletion of 10 of 18 genes of this class was reported to result in a synthetic growth defect in combination with *rsc7Δ*, indicating that the RSC complex shares a strong genetic relationship with the processes associated with these genes.

**Table 4:** Null mutations conferring growth defects in combination with *nps1-105*.

Classes^a^	Processes^a^	ORF	Gene^b^	Description^c^
Chromosome metabolism	Chromatin remodeling	*YNL059C*	*ARP5*	Actin-Related Protein
		*YOR141C*	*ARP8*	Actin-Related Protein
		*YLR357W*	*RSC2*	Remodel the Structure of Chromatin
		*YMR091C*	*NPL6/RSC7*	Nuclear Protein Localization
	Transcription	*YNR010W*	*CSE2*	Chromosome Segregation
		*YGR200C*	*ELP2*	ELongator Protein
		*YJL140W*	*RPB4*	RNA Polymerase B
		*YJR063W*	*RPA12*	RNA Polymerase A
		*YJL168C*	*SET2*	SET domain-containing
		*YCR084C*	*TUP1*	dTMP-Uptake
	DNA damage repair	*YOR258W*	*HNT3*	Histidine triad NucleoTide-binding
	Chromatid cohesion	*YHR191C*	*CTF8*	Chromosome Transmission Fidelity
	Spindle assembly checkpoint	*YGR188C*	*BUB1*	Budding Uninhibited by Benzimidazole
		*YDR532C*	*KRE28*	Subunit of a kinetochore-microtubule binding complex
	RNA processing	*YDR378C*	*LSM6*	Like Sm protein
		*YNL147W*	*LSM7*	Like Sm protein
		*YPR101W*	*SNT309*	Synthetic lethal to prp NineTeen mutation
	Nuclear pore	*YDL116W*	*NUP84*	NUclear Pore
Translation	Ribosome biogenesis	*YLR074C*	*BUD20*	BUD site selection
		*YCR047C*	*BUD23*	BUD site selection
		*YKR024C*	*DBP7*	Dead Box Protein
		*YGR271C-A*	*EFG1*	Exit From G1
		*YFR001W*	*LOC1*	LOCalization of ASH1 mRNA
		*YGR159C*	*NSR1*	Nucleolar protein that binds nuclear localization sequences
		*YMR142C*	*RPL13B*	Ribosomal Protein of the Large subunit
		*YHR010W*	*RPL27A*	Ribosomal Protein of the Large subunit
		*YDL075W*	*RPL31A*	Ribosomal Protein of the Large subunit
		*YJL189W*	*RPL39*	Ribosomal Protein of the Large subunit
		*YHR021C*	*RPS27B*	Ribosomal Protein of the Small subunit
	Regulation of translation	*YKL204W*	*EAP1*	EIF4E-Associated Protein
		*YGR162W*	*TIF4631*	Translation Initiation Factor
		*YOR302W*		Arginine attenuator peptide, regulates translation of the CPA1 mRNA
Mitochondria	Mitochondrial translation	*YLR069C*	*MEF1*	Mitochondrial Elongation Factor
		*YNL005C*	*MRP7*	Mitochondrial Ribosomal Protein
		*YLR439W*	*MRPL4*	Mitochondrial Ribosomal Protein, Large subunit
		*YBR282W*	*MRPL27*	Mitochondrial Ribosomal Protein, Large subunit
		*YCR003W*	*MRPL32*	Mitochondrial Ribosomal Protein, Large subunit
		*YPR100W*	*MRPL51*	Mitochondrial Ribosomal Protein, Large subunit
		*YBR251W*	*MRPS5*	Mitochondrial Ribosomal Protein, Small subunit
		*YPR047W*	*MSF1*	Mitochondrial aminoacyl-tRNA Synthetase, Phenylalanine (F)
		*YPL097W*	*MSY1*	Mitochondrial aminoacyl-tRNA Synthetase, tyrosine (Y)
		*YJR113C*	*RSM7*	Ribosomal Small subunit of Mitochondria
		*YNR037C*	*RSM19*	Ribosomal Small subunit of Mitochondria
	Mitochondria-nucleus	*YOL067C*	*RTG1*	ReTroGrade regulation
	retrograde regulation	*YGL252C*	*RTG2*	ReTroGrade regulation
		*YBL103C*	*RTG3*	ReTroGrade regulation
	Mitochondrial RNA processing	*YGR150C*	*CCM1*	COB and COX1 mRNA maturation
		*YIR021W*	*MRS1*	Mitochondrial RNA Splicing
	Mitochondrial genome	*YBR194W*	*AIM4*	Altered Inheritance rate of Mitochondria
	maintenance	*YBR179C*	*FZO1*	FuZzy Onions homolog
		*YDL198C*	*GGC1*	GDP/GTP Carrier
		*YJR144W*	*MGM101*	Mitochondrial Genome Maintenance
	Mitochondrial enzyme	*YAL044C*	*GCV3*	GlyCine cleaVage
		*YOR136W*	*IDH2*	Isocitrate DeHydrogenase
		*YBR221C*	*PDB1*	Pyruvate Dehydrogenase Beta subunit
		*YPL188W*	*POS5*	PerOxide Sensitive
		*YMR267W*	*PPA2*	PyroPhosphatAse
		*YJR104C*	*SOD1*	SuperOxide Dismutase
General metabolism	Amino acid biosynthesis	*YLR027C*	*AAT2*	Aspartate AminoTransferase
		*YJL071W*	*ARG2*	ARGinine requiring
		*YJL088W*	*ARG3*	ARGinine requiring
		*YER069W*	*ARG5*,*6*	ARGinine requiring
		*YDR127W*	*ARO1*	AROmatic amino acid requiring
		*YOR303W*	*CPA1*	Carbamyl Phosphate synthetase A
		*YAL012W*	*CYS3*	CYStathionine gamma-lyase
		*YEL046C*	*GLY1*	GLYcine requiring
		*YDR158W*	*HOM2*	HOMoserine requiring
		*YER052C*	*HOM3*	HOMoserine requiring
		*YJR139C*	*HOM6*	HOMoserine requiring
		*YHL011C*	*PRS3*	PhosphoRibosylpyrophosphate Synthetase
		*YOR184W*	*SER1*	SERine requiring
		*YGR208W*	*SER2*	SERine requiring
		*YCR053W*	*THR4*	THReonine requiring
	Carbohydrate	*YBR126C*	*TPS1*	Trehalose-6-Phosphate Synthase
Transport	Endosomal transport,	*YJL204C*	*RCY1*	ReCYcling
	protein targeting	*YDR137W*	*RGP1*	Reduced Growth Phenotype
		*YLR039C*	*RIC1*	RIbosome Control
		*YLR025W*	*SNF7*	Sucrose NonFermenting
		*YPL002C*	*SNF8*	Sucrose NonFermenting
		*YCL008C*	*STP22*	STerile Pseudoreversion
		*YBR127C*	*VMA2*	Vacuolar Membrane Atpase
		*YEL027W*	*VMA3*	Vacuolar Membrane Atpase
		*YJR102C*	*VPS25*	Vacuolar Protein Sorting
		*YPL065W*	*VPS28*	Vacuolar Protein Sorting
	Other transporter	*YHL047C*	*ARN2*	AFT1 ReguloN
		*YHR060W*	*VMA22*	Protein that is required for vacuolar H+-ATPase (V-ATPase) function
		*YKL119C*	*VPH2*	Vacuolar pH
Other	Autophagy	*YLR423C*	*ATG17*	AuTophaGy related
		*YLR240W*	*VPS34*	Vacuolar Protein Sorting
	Protein folding	*YLR244C*	*MAP1*	Methionine AminoPeptidase
	/targeting/maturation	*YNL064C*	*YDJ1*	Yeast dnaJ
	Cytoskeleton organization	*YPL161C*	*BEM4*	Bud Emergence
	Protein phosphatase	*YDR028C*	*REG1*	REsistance to Glucose repression
		*YBL058W*	*SHP1*	Suppressor of High-copy PP1
		*YDL047W*	*SIT4*	Suppressor of Initiation of Transcription

a) "Classes" and "Processes" of each gene are assigned manually based on SGD (www.yeastgenome.org).

b) Underlined genes were previously identified by an SGA analysis using *rsc7Δ* as a query [22].

c) Brief descriptions of each gene product are derived from the "Name description" in SGD.

A characteristic feature of the genes identified during our screening, compared with those identified in the previous study with *rsc7Δ* was the presence of genes within the second “translation” class, particularly those involved in ribosome biogenesis, and within the “mitochondria” class. An earlier genome-wide localization study of RSC revealed that this complex frequently localizes adjacent to RNA polymerase III (Pol III)-transcribed genes [23], and transcription of the Pol III-transcribed genes *SNR6* and *RPR1* was reported to be significantly reduced in Rsc4 C-terminal mutant cells [24]. Furthermore, recent studies showed that RSC depletion causes a pronounced decrease in Pol III occupancy and affects nucleosome density [25, 26]. These observations indicate that reduced Pol III-transcribed gene transcription in *nps1-105*, in combination with the deletion of genes that function in ribosome biogenesis or translation regulation, might have led to synthetic lethality/slow growth. In contrast, the relationships between RSC and genes implicated in mitochondrial function have not yet been well studied. Therefore, we focused on the mitochondrial functions of RSC.

### 2. *rsc* mutants exhibit mitochondrial function-defective phenotypes

**Fig 1:**
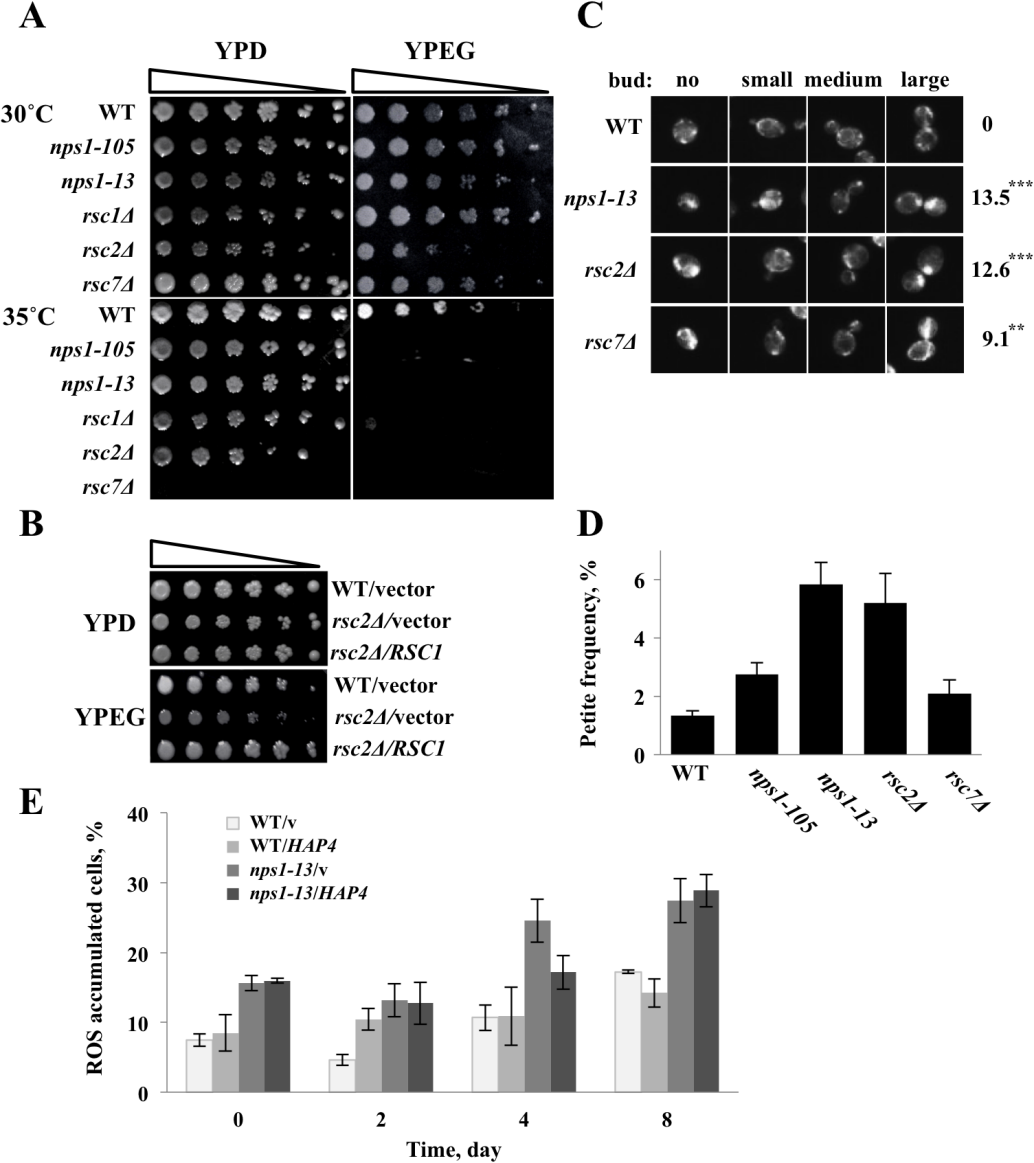
*rsc* mutants exhibit phenotypes defective in mitochondrial function. **(A)** *rsc* mutants exhibit growth defects on medium containing a non-fermentable carbon source. Five-fold serial dilutions of individual strains (WT (BY4743), *nps1-105* (BYI-17), *nps1-13* (BYI-3), *rsc1Δ* (BYI-1), *rsc2Δ* (BYI-2), and *rsc7Δ* (BYI-18)) were grown to log phase in YPD medium, spotted on YPD and YPEG plates, and incubated at the indicated temperatures for 3 days. **(B)** Overexpression of *RSC1* suppresses the growth defect of *rsc2Δ* on YPEG. Five-fold serial dilutions of exponentially growing individual strains (WT (BY4743) carrying YEp13 (WT/vector) and *rsc2Δ* (BYI-2) carrying YEp13 (*rsc2Δ*/vector) or YEp13*RSC1*-3MYC (*rsc2Δ/RSC1*)) were spotted on YPD and YPEG plates and incubated at 30°C for 3 days. **(C)** *rsc* mutants contain mitochondria with irregular morphologies. WT (BY4743), *nps1-13* (BYI-3), *rsc2Δ* (BYI-2), and *rsc7Δ* (BYI-18) cells were grown to log phase in YPD medium, stained with Mito-Tracker, and observed under a fluorescence microscope. Numerals on the right sides of panels represent the percentages of cells containing aggregated mitochondria among total cells. All *P*-values were calculated using the two-tailed chi-square test (≥50 cells; **P < 0.05, ***P < 0.005). **(D)** *rsc* mutations enhance mitochondrial DNA loss. WT (BY4743), *nps1-105* (BYI-17), *nps1-13* (BYI-3), *rsc2Δ* (BYI-2), and *rsc7Δ* (BYI-18) cells were plated on YPEG medium; three independent colonies were later picked and separately grown to stationary phase in YPD medium. Two hundred cells from each culture were plated on YPD plates and incubated at 30°C for 3 days. To assess the frequency of petite cells, we counted the total number of cells and the number of petite cells on each plate. Data are presented as the means ± SEM of three replicates. **(E)** *nps1-13* cells accumulate reactive oxygen species. WT (BY4743) and *nps1-13* (BYI-3) cells harboring pRS426 (WT/v and *nps1-13*/v, respectively) or pRS426*GPDpr*::*HAP4* (WT/*HAP4* and *nps1-13/HAP4*, respectively) were grown to log phase in SD-Ura medium, shifted to YPEG medium, and incubated at 30°C with shaking. On the indicated days, portions of the cells were separated, stained with dihydroethidium, and examined under a fluorescence microscope. The experiment was repeated three times (*n* = 300). Data are presented as the means ± SEM.

To investigate whether RSC mutations affected respiratory growth, we examined the growth of *rsc* mutant strains on a rich medium containing a non-fermentable carbon source (i.e., ethanol and glycerol (YPEG)). To confirm that these phenotypes were not specific to the *nps1-105*-mutated allele, we employed another temperature-sensitive mutant, *nps1-13* [14], and deletion mutants of *RSC1*, *RSC2*, and *RSC7* in this experiment. *nps1-13* contains amino-acid substitutions in the C-terminal bromodomain of Nps1, resulting in reduced interactions between RSC components. In this mutant cells, the existence of functional RSC complexes was estimated to be five times lower than in WT cells [14]. Rsc1 and Rsc2 are highly homologous proteins contained in distinct RSC complexes. Deletion of either *RSC1* or *RSC2* does not affect viability, but double deletion of these genes is lethal. The quantity of Rsc2 is 10-fold higher than that of Rsc1 [27]. As shown in Fig 1A, *nps1-13* and *rsc2Δ* cells exhibited impaired growth on a YPEG plate at 30°C. At 35°C, a semi-permissive temperature for growth of all evaluated *rsc* mutants except *rsc7Δ* on YPD, none of these *rsc* mutants grew on the YPEG plate. The data suggest that these *rsc* mutations induced a defect(s) in respiratory growth. Among the five *rsc* mutants used in this experiment, *rsc2Δ* cells exhibited the most severe growth defect on YPEG, suggesting a functional difference between Rsc1-containing (Rsc1-RSC) and Rsc2-containing RSC (Rsc2-RSC) complexes in respiratory growth regulation, with Rsc2-RSC playing a major role in this process. To examine this point, we over-expressed *RSC1* in *rsc2Δ*, and then assessed cell growth on the YPEG plate. As shown in Fig 1B, on the YPEG plate, the growth of *rsc2Δ* cells harboring *RSC1* in a high-copy vector was indistinguishable from that of WT cells, suggesting that RSC complexes containing either Rsc1 or Rsc2 act redundantly to regulate respiratory growth.

Next, we used fluorescence microscopy to observe mitochondrial morphology in RSC mutants stained with Mito-Tracker (Fig 1C). In WT cells, mitochondria appeared as tubular networks distributed near the cell cortex. In contrast, mitochondria in *nps1-13* and *rsc2Δ* cells frequently aggregated to form one or two spots. Mitochondrial aggregation was observed in the cells at all cell-cycle stages. The frequencies of *nps1-13* and *rsc2Δ* cells containing aggregated mitochondria were 13.5% and 12.6%, respectively. Aggregated mitochondria were also observed in *rsc7Δ* cells, albeit at a lower frequency (9.1%). In contrast, little aggregation was observed in WT cells (<10^−4^).

The yeast mitochondrial genome is subject to spontaneous mutations that result in a loss of mitochondrial DNA (mtDNA). Cells that have lost mtDNA form small colonies, termed “petite”, on YPD medium. A defect in respiratory function is known to enhance mtDNA loss. To examine the stability of mtDNA in the *rsc* mutant cells, we measured the frequencies of petite formation in each strain cultured in YPD to the early-stationary phase. As shown in Fig 1D, all *rsc* mutants formed petite colonies at higher frequencies than did WT cells; especially, the petite frequencies of *nps1-13* and *rsc2Δ* were three-fold to four-fold higher than that of WT cells, indicating that the defective RSC complex induced mitochondrial genome instability.

These results indicate that the RSC complex plays important roles relevant to mitochondrial respiratory function. Mitochondrial dysfunction also leads to the accumulation of reactive oxygen species (ROS). As shown in Fig 1E, the rate of ROS accumulation was approximately two-fold higher in *nps1-13* cells than in WT cells. Among the *rsc* mutants used in this study, *nps1-13* and *rsc2Δ* exhibited the most severe phenotypes. Given that the functional RSC contents in these strains were estimated to be approximately 5-fold to 10-fold lower than those in WT cells, the former may require a larger amount of RSC under respiratory conditions relative to fermentation conditions.

### 3. Global transcription analysis of *nps1-13* grown under respiratory conditions

**Fig 2:**
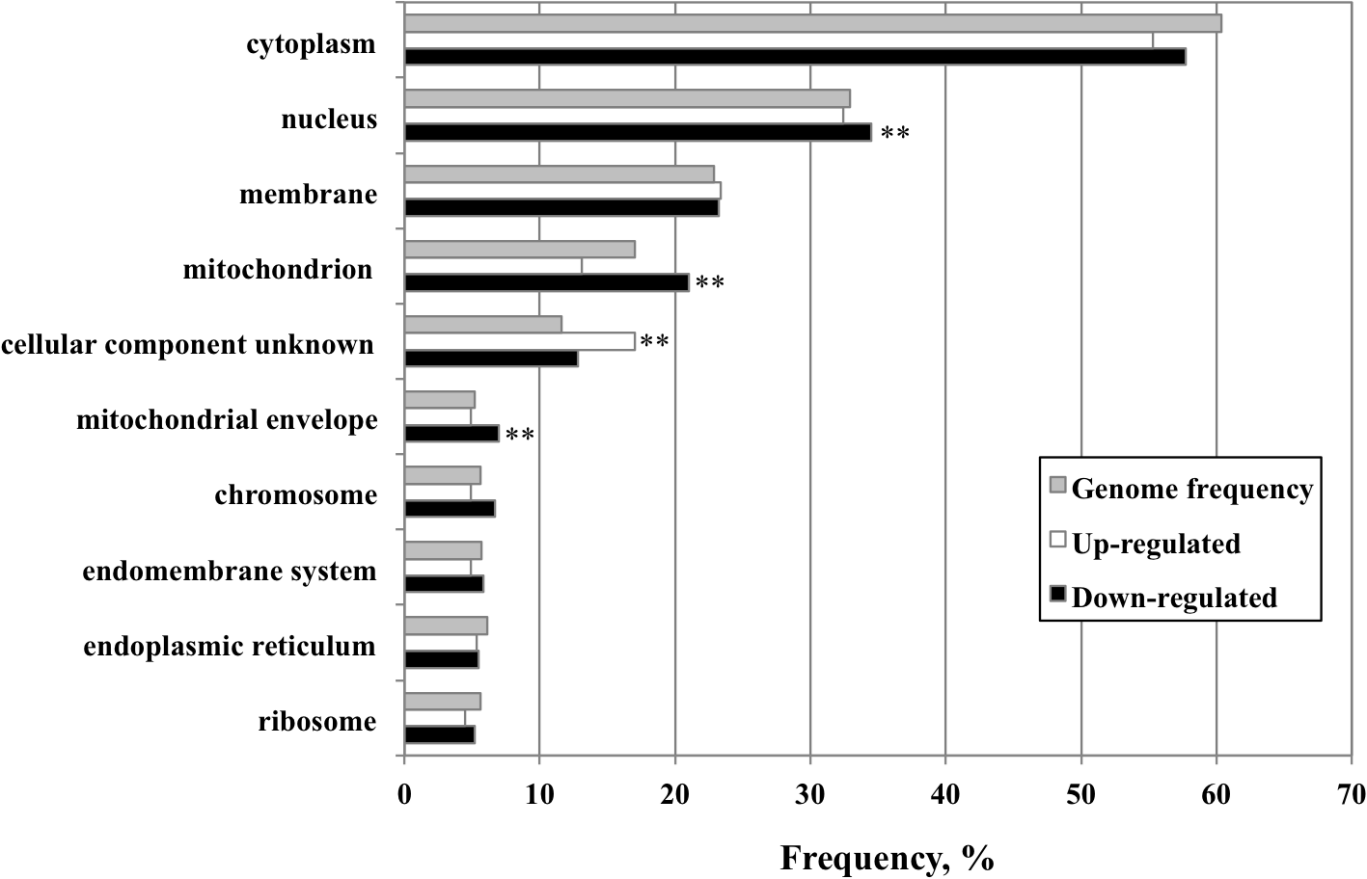
Comparison of genes up- or down-regulated genes in *nps1-13*, grouped using the GO Slim-Mapper with respect to cellular component. Only the GO terms that appeared in more than 6% of the up- and down-regulated genes are listed. For the remaining GO terms, no significant differences were observed between the frequencies of affected genes and the general frequency. All *P*-values were obtained using the hypergeometric test (**P < 0.05).

To further understand the function of RSC in respiratory growth, we performed a microarray analysis to compare global gene expression profiles between WT and *nps1-13* mutant cells grown on YPEG. From valid data on 5,558 genes in WT and *nps1-13* cells, 219 and 345 genes in *nps1-13* were found to be up- and down-regulated, respectively, above or below the two-fold threshold (S3 Table). A GO Slim-Mapper analysis of these up- or down-regulated genes revealed that the frequencies of down-regulated genes in the mitochondrion and mitochondrial envelope categories were significantly higher than the general frequency (*P*-values = 0.022 and 0.029, respectively; Fig 2). This result suggests that RSC is required for the expression of these genes. The mitochondrion-related genes with down-regulated expression in *nps1-13* are listed in Table 5. Of these 71 genes, 14 were grouped in the “respiration” category and considered closely relevant to the major phenotypes of *rsc* mutants described in the previous section. It was especially interesting to find that 7 of these 14 respiration genes were targets of the HAP complex (*P*-value = 0.038). This HAP complex is a transcription factor composed of Hap2, Hap3, Hap4, and Hap5 and plays a pivotal role in respiratory gene regulation [10]. In addition to the “respiration” group genes, *UPS2* in the “organization” group and *YMR31* in the “translation” group were also defined as Hap4 target genes. Moreover, although the *DNM1*, *MRPS5*, and *MEF1* genes, indicated with asterisks in Table 5, do not contain the Hap4-binding sequence in their promoters, expression of these genes has been reported to be controlled by the HAP complex [21]. These results suggest the possibility that RSC might act with the HAP complex to regulate its target genes.

**Table 5:** Functional grouping of mitochondria-related genes down-regulated in *nps1-13*.

ORF	Gene	logFC(*nps1-13*/WT)	Description
***Respiration***			
*YBL045C*	*COR1*	-1.396528328	CORe protein of QH2 cytocrome c reductase
*YBL099W*	*ATP1*	-1.704958554	ATP synthase
*YDL004W*	*ATP16*	-2.557231151	ATP synthase
*YDR079W*	*PET100*	-1.019022029	PETite colonies
*YGL018C*	*JAC1*	-1.038732523	J-type Accessory Chaperone
*YGR029W*	*ERV1*	-1.109214546	Essential for Respiration and Viability
*YGR101W*	*PCP1*	-1.703295695	Processing of Cytochrome c Peroxidase
*YHR051W*	*COX6*	-1.230934459	Cytochrome c OXidase
*YKL055C*	*OAR1*	-1.606497792	3-Oxoacyl-[Acyl-carrier-protein] Reductase
*YLR038C*	*COX12*	-1.257634276	Cytochrome c OXidase
*YLR164W*	*SHH4*	-1.286480017	SDH4 Homolog
*YLR295C*	*ATP14*	-1.136379747	ATP synthase
*YMR267W*	*PPA2*	-1.556696229	PyroPhosphatAse
*YPL270W*	*MDL2*	-1.283689019	MultiDrug resistance-Like
***Metabolism***			
*YCL064C*	*CHA1*	-1.326318344	Catabolism of Hydroxy Amino acids
*YDR305C*	*HNT2*	-1.300467492	Histidine triad NucleoTide-binding
*YER019W*	*ISC1*	-1.104381351	Inositol phosphoSphingolipid phospholipase C
*YER069W*	*ARG5*,*6*	-1.052067786	ARGinine requiring
*YER183C*	*FAU1*	-1.260016198	Folinic Acid Utilization
*YFL030W*	*AGX1*	-1.11633377	Alanine:Glyoxylate aminotrans(X)ferase
*YGL059W*	*PKP2*	-1.169211802	Protein Kinase of PDH
*YGR102C*	*GTF1*	-3.704929047	Glutaminyl Transamidase subunit F
*YGR171C*	*MSM1*	-1.09387038	Mitochondrial aminoacyl-tRNA Synthetase, Methionine
*YJL005W*	*CYR1*	-1.436163151	CYclic AMP Requirement
*YJL130C*	*URA2*	-1.171207794	URAcil requiring
*YJR051W*	*OSM1*	-1.664915789	OSMotic sensitivity
*YKL094W*	*YJU3*	-1.339639536	Monoglyceride lipase (MGL)
*YLL027W*	*ISA1*	-1.608964228	Iron Sulfur Assembly
*YMR002W*	*MIC17*	-1.27657268	Mitochondrial Intermembrane space Cysteine motif protein
*YNL009W*	*IDP3*	-1.957974411	Isocitrate Dehydrogenase, NADP-specific
*YNL104C*	*LEU4*	-1.326612984	LEUcine biosynthesis
*YNL318C*	*HXT14*	-1.082656945	HeXose Transporter
*YOL045W*	*PSK2*	-1.053181566	Pas domain-containing Serine/threonine protein Kinase
*YPL091W*	*GLR1*	-1.926695216	Cytosolic and mitochondrial glutathione oxidoreductase
***Organigation***			
*YBR179C*	*FZO1*	-1.630212553	FuZzy Onions homolog
*YHR194W*	*MDM31*	-1.336269739	Mitochondrial Distribution and Morphology
*YIL062C*	*ARC15*	-1.22799372	ARp2/3 Complex subunit
YLL001W*	DNM1*	-2.739316701	DyNaMin-related
*YLR168C*	*UPS2*	-1.281524891	UnProceSsed
*YNL026W*	*SAM50*	-1.00279983	Sorting and Assembly Machinery
*YPL029W*	*SUV3*	-1.011994907	SUppressor of Var1
***Chromosome metabolism***			
*YDL164C*	*CDC9*	-1.486943089	Cell Division Cycle
*YKL113C*	*RAD27*	-1.62562909	RADiation sensitive
*YMR167W*	*MLH1*	-1.615848689	MutL Homolog
*YOL042W*	*NGL1*	-1.252177233	Putative endonuclease
*YPL155C*	*KIP2*	-1.646124618	KInesin related Protein
***Translation***			
YBR251W*	MRPS5*	-1.148985411	Mitochondrial Ribosomal Protein, Small subunit
*YDR077W*	*SED1*	-1.621599493	Suppression of Exponential Defect
*YER153C*	*PET122*	-1.221689033	PETite colonies
*YFR049W*	*YMR31*	-1.09117704	Yeast Mitochondrial Ribosomal protein
*YHR070W*	*TRM5*	-1.201399879	Transfer RNA Methyltransferase
*YHR189W*	*PTH1*	-1.133275954	Peptidyl-Trna Hydrolase
YLR069C*	MEF1*	-1.015712709	Mitochondrial Elongation Factor
*YMR158W*	*MRPS8*	-1.052102798	Mitochondrial Ribosomal Protein, Small subunit
*YNL227C*	*JJJ1*	-1.094508612	J-protein (Type III)
*YOL141W*	*PPM2*	-1.031449449	Protein Phosphatase Methyltransferase
*YOR048C*	*RAT1*	-1.205794524	Ribonucleic Acid Trafficking
*YOR188W*	*MSB1*	-1.4223978	Multicopy Suppressor of a Budding defect
*YOR335C*	*ALA1*	-1.034833773	ALAnyl-tRNA synthetase
*YPL005W*	*AEP3*	-1.086709933	ATPase ExPression
*YPL082C*	*MOT1*	-1.191021182	Modifier of Transcription
***Other***			
*YDL040C*	*NAT1*	-1.987231644	N-terminal AcetylTransferase
*YLR090W*	*XDJ1*	-1.238779632	Putative chaperone
*YOL109W*	*ZEO1*	-1.128791113	ZEOcin resistance
*YPR095C*	*SYT1*	-1.015582931	Suppressor of ypt3
***Unknown***			
*YGR021W*		-2.127560354	Putative protein of unknown function
*YHL014C*	*YLF2*	-1.662532462	protein of unknown function
*YKR070W*		-1.259135277	Putative protein of unknown function
*YMR221C*	*FMP42*	-1.013995342	Found in Mitochondrial Proteome
*YNL122C*		-2.052330445	Putative protein of unknown function
*YPR097W*		-1.622035952	Protein that contains a Phox homology (PX) domain

Underlined genes are target of transcription factor HAP complex. Genes with asterisk do not contain HAP complex binding site, but their expression is under the control of the complex. [21]

**Fig 3:**
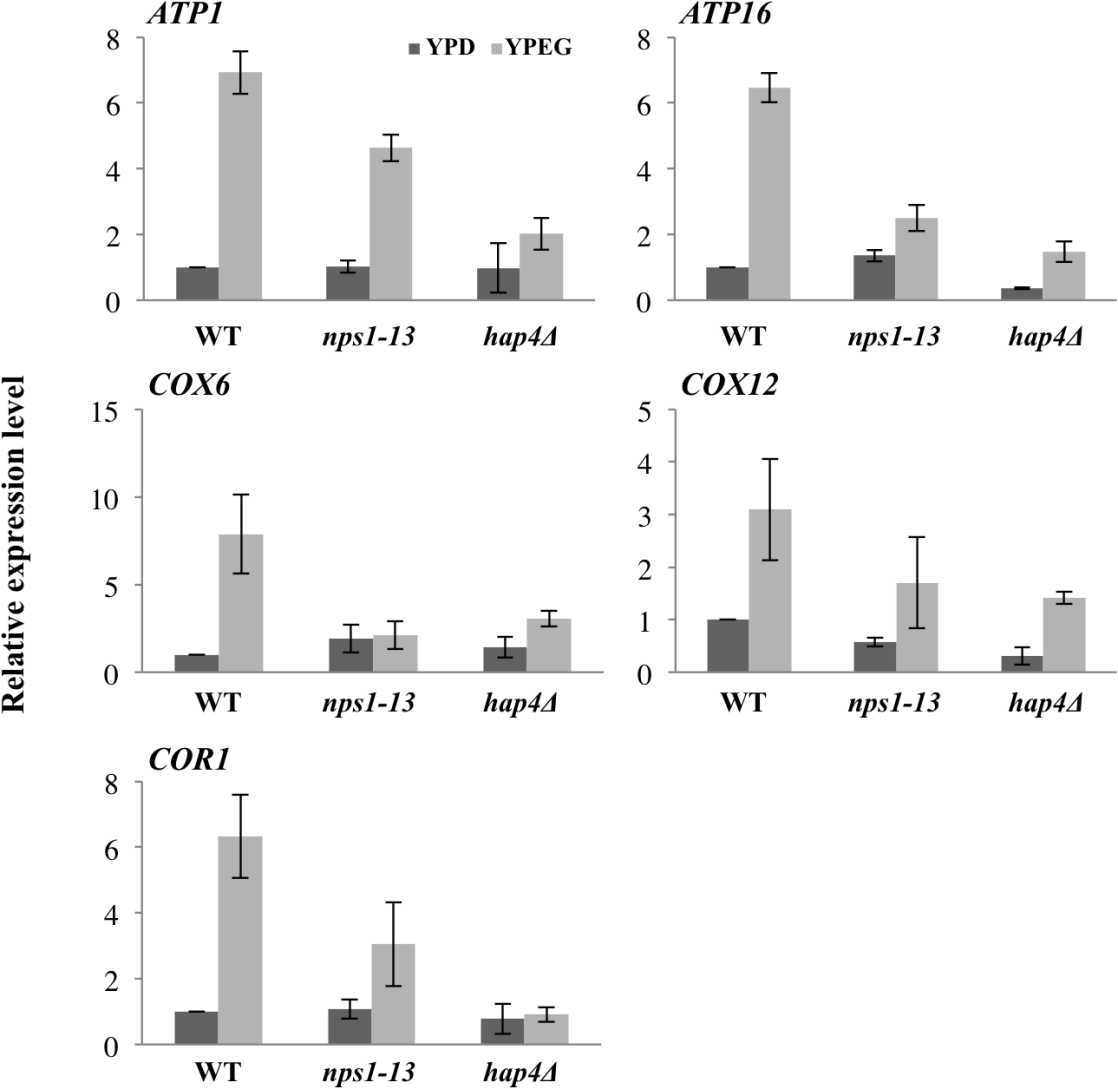
Gene expression analysis. Relative gene expression levels of *ATP1*, *ATP16*, *XOX1*, *COX12*, and *COR1* were measured via quantitative PCR using RNA isolated from WT (BY4743), *nps1-13* (BYI-3), and *hap4Δ* (BYI-22) cells grown in YPD or YPEG. The mRNA level of each gene was normalized to that of *ACT1*, and is indicated as relative to the value for WT cells grown in YPD, which was set at “1”. Data are presented as the mean ± SEM (*n* = 3).

To validate these microarray results, we performed quantitative real-time PCR for HAP complex target genes, *ATP1*, *ATP16*, *COR1*, *COX6*, and *COX12*. Consistent with the array data, induction of these genes in *nps1-13* during growth on YPEG was lower than that observed in WT cells (Fig 3).

### 4. RSC interacts with Hap4 to regulate the expression of respiratory genes

**Fig 4:**
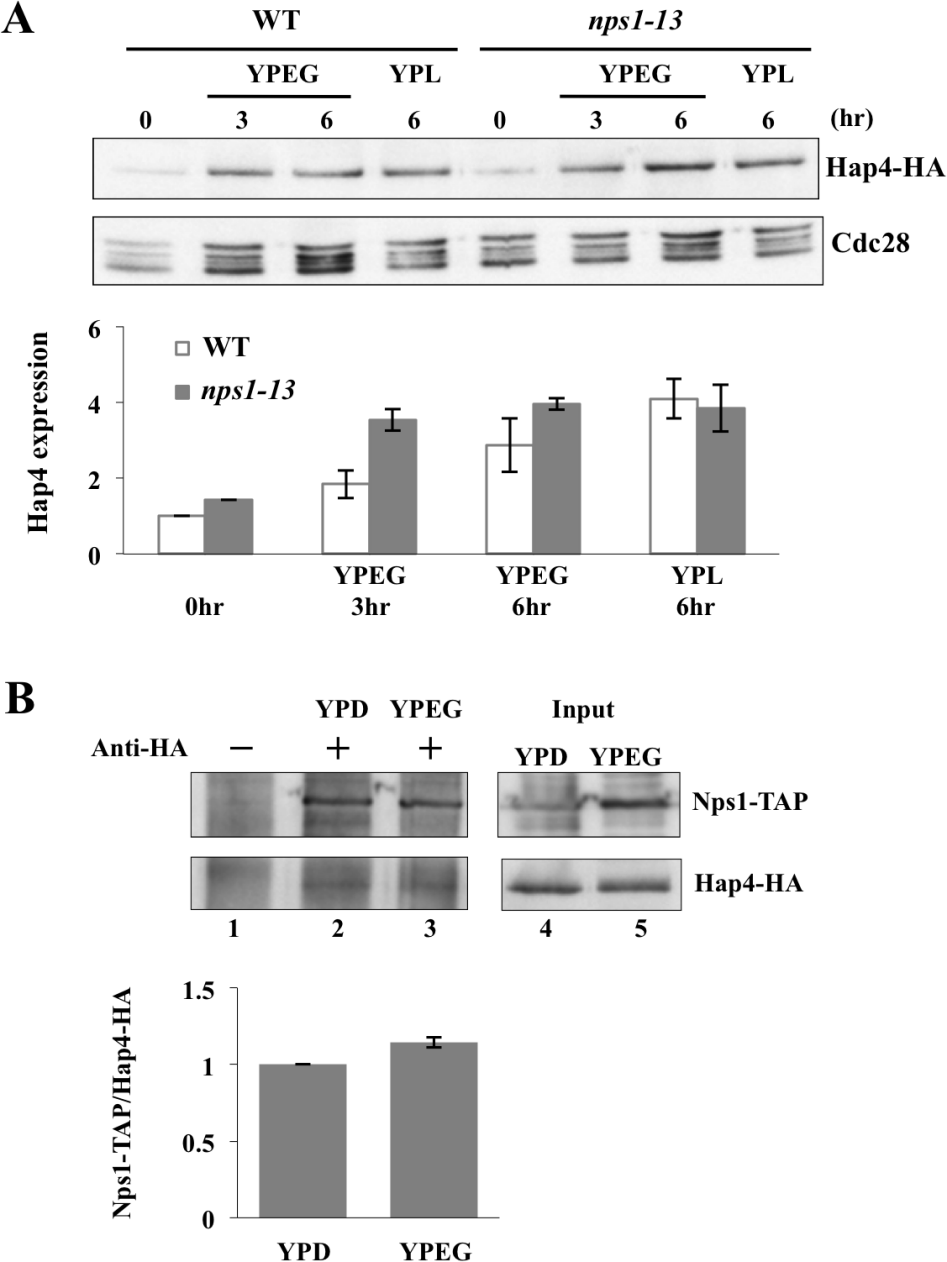
Nps1 physically interacts with Hap4. **(A)** *nps1-13* mutation does not affect Hap4 expression. *HAP4-HA* (BYI-19) and *nps1-13 HAP4-HA* (BYI-21) cells were grown to log phase in YPEG or YPL medium for the times indicated in the figure, after which whole-cell extracts were prepared. Proteins in the extract were separated by SDS-PAGE, and Hap4-HA was detected by immunoblotting. The densities of immunoblot bands labeled with anti-HA were normalized to those labeled with anti-Cdc28 and indicated as a bar graph of values relative to the value of WT cells grown for 0 h in YPD, which was set at “1”. Data are presented as the means ± SEM (*n* = 3). **(B)** Nps1-TAP physically interacts with Hap4-HA. BYI-20 (*NPS1-TAP-KanMX4 HAP4-6HA*::*URA3*) cells were grown to log phase in YPD medium and subsequently shifted to YPEG medium, where they were maintained for 3 h. Immunoprecipitates prepared from cell lysates with anti-HA antibody were subjected to immunoblotting with anti-TAP and anti-HA antibodies. The densities of immunoblot bands stained with anti-TAP in lanes 2 and 3 were normalized with those of bands stained with anti-HA and indicated as a bar graph of values relative to the value of YPD, which was set at “1”. Data are presented as the means ± SEM (*n* = 3).

The reduced expression of HAP-regulated genes in *nps1-13* suggested the possibility that this mutation affected Hap4 expression because HAP complex activity is proportional to the Hap4 subunit level [28]. To verify the level of Hap4, we constructed strains expressing HA-tagged Hap4 and examined the Hap4-HA content by Western blotting. As described previously, the Hap4 level increases upon shifting cells from a medium containing glucose to medium containing a non-fermentable carbon source [10]. This induction was not affected by the *nps1-13* mutation (Fig 4A). Next, we examined whether Nps1 physically interacted with Hap4 by performing a co-immunoprecipitation experiment using a strain expressing both Nps1-TAP and Hap4-HA. As indicated in Fig 4B, Nps1-TAP was detected in immunoprecipitates prepared using an anti-HA antibody, indicating that Nps1-TAP and Hap4-HA physically interacted *in vivo*. These results suggest that RSC might function together with the HAP complex to induce a set of respiratory genes. However, it is also possibile that each complex is independently recruited to the promoter of a target gene through an interaction with some other factor(s) such as histone acetyltransferase or histone deacetylase. Further analysis is required to understand the mechanisms of gene regulation.

**Fig 5:**
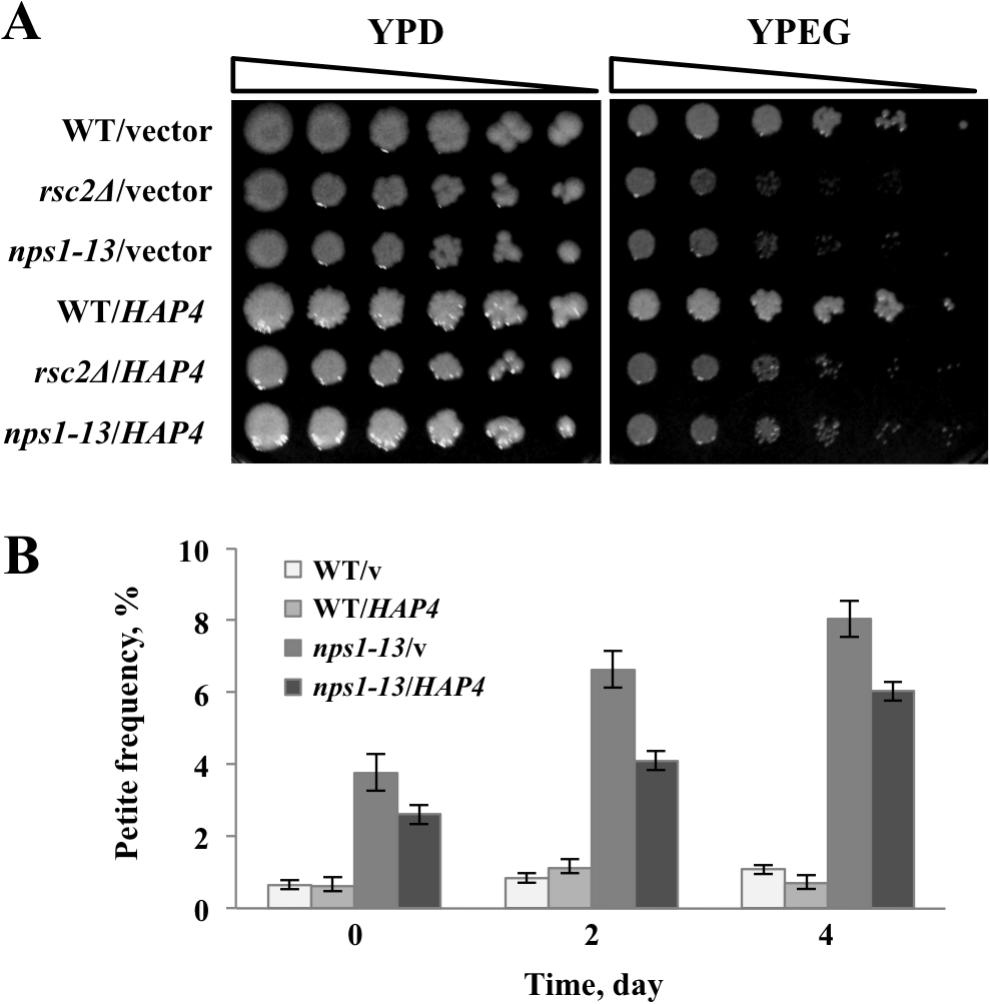
Overexpression of *HAP4* alleviated the respiratory defect of *nps1-13*. **(A)** Effect of *HAP4* overexpression on *nps1-13* growth on YPEG. WT (BY4743) and *nps1-13* (BYI-3) cells harboring pRS426 (WT/v and *nps1-13*/v, respectively) or pRS426*GPDpr*::*HAP4* (WT/*HAP4* and *nps1-13/HAP4*, respectively) were grown to log phase in SD-Ura medium, spotted on YPD and YPEG plates in serial five-fold dilutions and incubated at 30°C for 3 days. **(B)** Effect of *HAP4* overexpression on petit *nps1-13* colony formation. The strains described in **(A)** were plated on YPEG; three independent colonies were subsequently picked, pre-cultured in SD-Ura medium, and separately grown in YPD medium. On the indicated days, 200 cells from each culture were plated on YPD plates and incubated at 30°C for 3 days. Data are presented as the means ± SEM (*n* = 3).

To understand the relationship between the RSC and HAP complex, we first determined whether the *hap4Δ* mutant exhibits similar phenotypes as those induced by mitochondrial dysfunction in *rsc* mutants. The *hap4Δ* mutant also exhibited growth defects on a medium containing a non-fermentable carbon source and a high frequency of mtDNA loss (S1 Fig). Next, we examined whether the respiratory defect phenotypes of *rsc* mutants could be relieved by the overexpression of *HAP4*. For this experiment, we constructed a high-copy plasmid carrying *HAP4* that was expressed under the control of the *GPD* promoter (pRS426*GPDpr*::*HAP4*). As shown in Fig 5A and 5B, defective growth of *nps1-13* and *rsc2Δ* on YPEG plates and enhanced formation of petite *nps1-13* colonies were alleviated by the overexpression of *HAP4*. In contrast, little recovery was observed with regard to the increased accumulation of ROS in *nps1-13* cells (Fig 1E). These results suggest the involvement of RSC in the transcriptional activation of a set of respiratory genes, together with the HAP complex. However, the results also indicate that RSC might interact with factors other than the HAP complex to regulate mitochondrial function.

In conclusion, our results are the first to show the relevance of the RSC to mitochondrial function. Our findings show that cells require higher amounts of RSC under respiratory condition, compared with fermentable condition, indicating that RSC may orchestrate the expression of genes required for mitochondrial function together with transcription factors other than the HAP complex. Identification of these factors should elucidate the regulation of respiration and mitochondrial development. Mitochondrial dysfunction has been linked to a range of pathologies, including cancer (reviewed in [29]). As described, high frequencies of mutations in the components of human chromatin-remodeling complexes have been identified in human cancers; however, the molecular mechanisms underlying the carcinogenic effects of these mutations are largely unknown [30, 31]. In this context, further analysis of the effects of RSC on mitochondrial function should facilitate a better understanding of the functions of mammalian ATP-dependent chromatin remodelers in carcinogenesis.

## Supporting Information

S1 Fig *hap* mutant exhibits phenotypes caused by mitochondrial dysfunction. **(A)** A *hap4Δ* mutant exhibits growth defect on medium containing a non-fermentable carbon source. Five-fold serial dilutions of individual strains (WT (BY4743), *hap4Δ* (BYI-22), and *nps1-13* (BYI-3)) were grown to log phase in YPD medium, spotted on YPD and YPEG plates, and incubated at the indicated temperatures for 3 days. **(B)** *hap4Δ* mutation enhances mitochondrial DNA loss. WT (BY4743), *hap4Δ* (BYI-22), and *nps1-13* (BYI-3) cells were plated on YPEG; three independent colonies were subsequently picked and grown separately in YPD medium to stationary phase. Two hundred cells from each culture were plated on YPD and incubated at 30°C for 3 days. To assess the frequency of petite colonies, we counted the total number of viable cells and the number of petite colonies on each plate. Data are presented as the means ± SEM of three replicates.
(TIF)

S1 Table Reproducibility of the SGA analysis. (XLSX)

S2 Table GO terms enriched among the 95 positive genes in terms of biological process. (XLSX)

S3 Table Differential DNA microarray data of *nps1-13* vs. BY4743. (XLSX)
