# Supplementary figures and images for: RSC Chromatin-Remodeling Complex Is Important for Mitochondrial Function in Saccharomyces cerevisiae
Source: PLoS One. 2015 Jun 18;10(6):e0130397. doi: 10.1371/journal.pone.0130397 (PMC4472808; doi:10.1371/journal.pone.0130397)

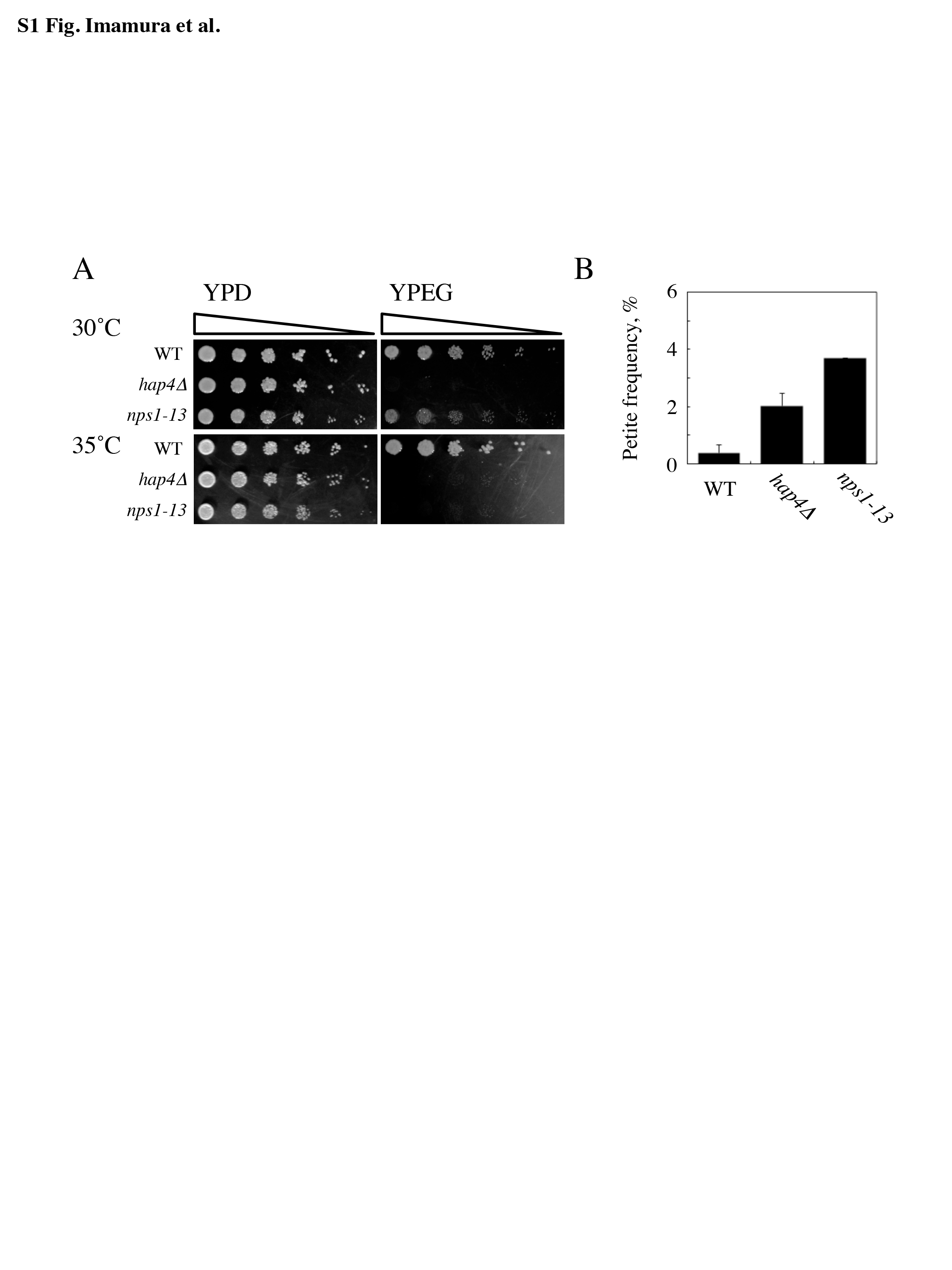

Supplement: S1 Fig — (A) A hap4Δ mutant exhibits growth defect on medium containing a non-fermentable carbon source. Five-fold serial dilutions of individual strains (WT (BY4743), hap4Δ (BYI-22), and nps1-13 (BYI-3)) were grown to log phase in YPD medium, spotted on YPD and YPEG plates, and incubated at the indicated temperatures for 3 days. (B) hap4Δ mutation enhances mitochondrial DNA loss. WT (BY4743), hap4Δ (BYI-22), and nps1-13 (BYI-3) cells were plated on YPEG; three independent colonies were subsequently picked and grown separately in YPD medium to stationary phase. Two hundred cells from each culture were plated on YPD and incubated at 30°C for 3 days. To assess the frequency of petite colonies, we counted the total number of viable cells and the number of petite colonies on each plate. Data are presented as the means ± SEM of three replicates. (TIF) [file pone.0130397.s001.tif]
